# Regulating growth and stress responses in *Abies beshanzuensis* seedlings: integrated impacts of shading on morphology, physiology, and molecular pathways

**DOI:** 10.3389/fpls.2026.1833397

**Published:** 2026-05-13

**Authors:** Hongfei Lu, Chuting Yan, Zhen Pang, Rongguang Lan, Lifang Zhang, Sumei Wu, Yougui Wu, Xiaoyi Li, Likang Zhao, Mingjian Yu

**Affiliations:** 1 Dongyang Wood Carving Industry Innovation Research Institute, Zhejiang Guangsha Vocational and Technical University of Construction, Dongyang, Zhejiang, China; 2 College of Life Science, Zhejiang Sci-Tech University, Zhejiang Province Key Laboratory of Plant Secondary Metabolism and Regulation, Hangzhou, China; 3 Qianjiangyuan-Baishan National Park Qinyuan Conservation Center, Qingyuan, China; 4 College of Life Science, Zhejiang University, Hangzhou, China

**Keywords:** antioxidant enzymes, molecular adaptations, oxidative stress, photosynthetic performance, shade treatments

## Abstract

**Introduction:**

*Abies beshanzuensis* M. H. Wu is a critically endangered conifer species of immense ecological and evolutionary significance, with only three wild adult progenitor trees remaining globally. Due to its extreme vulnerability to environmental stress, poor natural regeneration, and obligate mycorrhizal dependency, identifying optimal microclimatic conditions for artificial cultivation is paramount for its survival. This study investigates how varying shading intensities influence the morpho-architectural, physiological, and transcriptomic responses of *A. beshanzuensis* seedlings to establish ideal conservation protocols.

**Methods:**

Seedlings were subjected to multiple shading treatments arrayed along an intensity gradient, culminating in extreme shading at T5. The intermediate treatment, T3, represented moderate shading. The experimental framework evaluated phenotypic morphology (plant height, crown width, and root system architecture), physiological parameters (leaf chlorophyll content, chlorophyll fluorescence, antioxidant enzyme activities, and oxidative stress markers), and osmotic adjustments. Furthermore, global transcriptome profiling utilizing advanced RNA-Seq methodologies coupled with Gene Ontology (GO) and Kyoto Encyclopedia of Genes and Genomes (KEGG) enrichment analyses was conducted to identify Differentially Expressed Genes (DEGs) and map regulatory molecular pathways.

**Results:**

Moderate shading (T3) profoundly enhanced plant height, crown width, root system architecture, and leaf chlorophyll content, significantly outperforming all other treatments. Root and shoot growth initially increased with shading intensity but declined sharply under excessive shading (T4 and T5). Chlorophyll fluorescence analysis demonstrated optimized photosynthetic performance under T3, characterized by higher electron transport rates and maximal photochemical efficiency, whereas extreme shading (T5) suppressed these parameters. Physiological assessments revealed that T3 seedlings exhibited the highest antioxidant enzyme activities (Superoxide Dismutase, Peroxidase, Catalase) and the lowest Malondialdehyde content, indicating minimal oxidative membrane damage. Soluble sugar, protein, and proline levels fluctuated, reflecting adaptive metabolic adjustments. Transcriptome analysis identified extensive DEGs across treatments, with significant enrichment in pathways related to photosynthesis, secondary metabolism, stress responses, and hormonal signaling. GO and KEGG analyses confirmed that T3 optimized energy metabolism, enhanced defense mechanisms, and differentially regulated hormone signal transduction.

**Discussion:**

Moderate shading (T3) establishes highly favorable environmental conditions for *A. beshanzuensis* by achieving a critical biological equilibrium between photosynthetic efficiency, stress resilience, and molecular regulation. Excessive shading disrupts this balance, precipitating carbon starvation and physiological decline. These findings illuminate the underlying regulatory networks of shade adaptation and provide a vital theoretical and practical foundation for the ex-situ conservation, and artificial cultivation of this critically endangered relict species.

## Introduction

*Abies beshanzuensis* M.H.Wu is an evergreen coniferous relict tree distributed exclusively in Zhejiang, China (Hu et al., 2022). Morphologically, this species is a medium-sized coniferous tree characterized by irregularly fissured grey-white bark, opposite branches bearing linear, emarginate leaves with two abaxial white stomatal bands, and cylindrical cones that mature by November into pale yellow-brown strobili featuring fan-shaped seed scales, slightly exserted and reflexed bract scales, and winged seeds. Globally, only three wild trees of *A. beshanzuensi* remain, classifying the species as critically endangered (He, 2009). In 1987, the International Union for Conservation of Nature (IUCN) listed *A. beshanzuensis* among the twelve most critically endangered species worldwide (Surhone et al., 2011). Global warming poses a significant threat to the growth and survival of this species, highlighting the pressing need for comprehensive conservation of its germplasm resources, a thorough investigation into the mechanisms underlying its endangered status, and the formulation of effective strategies to expand its restricted habitat range. The survival of *A. beshanzuensis* seedlings is pivotal for population establishment, with light identified as a key environmental factor influencing their growth and development. Previous studies had showed that moderate shading increases the biomass accumulation of *Abies alba* (Grassi and Bagnaresi, 2001). However, no studies had reported the response of *A. beshanzuensis* to light stress. Therefore, studying the effects of varying light conditions on the critically endangered *A. beshanzuensis* is essential for understanding the physiological and molecular mechanisms driving seedling survival, thereby providing critical guidance for effective conservation, habitat expansion, and species recovery strategies.

External light conditions influence plant growth and development, with varying light intensities exerting distinct effects on their physiological traits. Excessive light energy impaired the functionality of photosystem I and photosystem II, reduced carbon assimilation, and damaged the structure and function of plant organs (Zheng et al., 2020). In severe cases, such stress caused plant mortality (Vialet-Chabrand et al., 2017). Conversely, insufficient light energy reduced photosynthetic rates below respiratory demands, leading to decreased organic matter accumulation and, eventually, reduced plant growth or mortality (Matuszyńska et al., 2019). Therefore, it was necessary to analyze the physiological conditions of plants under different light environments to infer the optimal growth conditions. In addition, appropriate shading increased the content of soluble sugars, soluble proteins, and proline in plant, thereby reducing the damage caused by light stress (Gao et al., 2019). Under shaded conditions, plant antioxidant enzymes (superoxide dismutase (SOD), peroxidase (POD), catalase (CAT), glutathione reductase, glutathione peroxidase, and ascorbate peroxidase) reduced lipid peroxidation damage by scavenging reactive oxygen species (Xu et al., 2024).

Light governs plant growth and metabolism by activating light-sensitive pigments, enhancing enzyme synthesis, and increasing carbohydrate content (Fang et al., 2022). Research highlighted that light intensity and quality regulated plant secondary metabolism (Zheng et al., 2019; Ye et al., 2021; Lin et al., 2021). Shading modulates the expression of genes associated with photosynthesis, primary metabolism, and reactive oxygen species scavenging in plants (Xu et al., 2024). These adjustments optimized photosynthesis, enhanced energy consumption of excess reductants, and minimized photooxidative stress. Light conditions influenced hormone biosynthesis and transport in plants (Zhao et al., 2024; Liu et al., 2024). In addition, Studies demonstrated that light altered the expression of auxin (IAA) signaling genes (Lv et al., 2021; Gai et al., 2025). Shading affected the expression of the gibberellin (GA) biosynthetic gene (*GA20ox*), thereby increasing the plant tolerance (Li et al., 2017). Under shading treatment, the levels of cytokinin, salicylic acid, and jasmonic acid were altered, which influenced plant morphogenesis (Kurepin et al., 2012; Muthu et al., 2016). However, the regulatory mechanism of *A. beshanzuensis* under shading treatment is still unknown.

Studies on plant shading revealed that shading treatment enhanced light utilization efficiency and energy metabolism flexibility (Gao et al., 2020). The plants demonstrated adaptive responses to shading by enhancing antioxidant enzyme activity and improving osmotic regulation. However, the specific adaptation and regulatory mechanisms of *Acer beshanzuensis* under shaded conditions remain poorly understood. This study explores the physiological and photosynthetic responses of *A. beshanzuensis* leaves to different light intensities. Comparative transcriptomic analysis revealed significant alterations in gene expression patterns under shading conditions. These findings provide valuable insights into the response mechanisms and physiological adaptations of *A. beshanzuensis* to shaded environments.

## Materials and methods

### Plant materials, growth conditions, and different light intensities

A total of 540 two-year-old seedling trees of A. beshanzuensis with similar growth years, size, and basal diameter were selected. The seedlings were transplanted to a nursery near Baisan Station (119°12’21”E, 27°45’5”N) with a spacing of 10 cm × 10 cm between plants. The region is characterized by an average annual temperature of 12.8 °C and an average annual precipitation of 2341.8 mm. The relative humidity averages 84.0% annually, while the recorded extreme temperatures range from a minimum of -13.2 °C to a maximum of 30.1 °C. Additionally, the frost-free period lasts for 187 days. At a height of approximately 1.5 meters on the A. beshanzuensis, nylon black nets with varying densities were applied to create different shading treatments. A light meter (GM1010, Bentech, Shenzhen, China) was used to measure light intensity on sunny days to determine the shading levels. The shading experiment included six treatments: Control (CK, natural sunlight), 15% shading (T1, transmitting 85% of natural sunlight), 30% shading (T2, transmitting 70% of natural sunlight), 45% shading (T3, transmitting 55% of natural sunlight), 60% shading (T4, transmitting 40% of natural sunlight), and 75% shading (T5, transmitting 25% of natural sunlight). The awning height was set at 1.5 m to optimize measurement and sampling conditions. A clearance of 20–30 cm from the ground was maintained to ensure proper ventilation, while sufficient spacing between adjacent awnings was provided to reduce interference. The 540 seedlings were randomly assigned to the six light intensity treatments, with 90 seedlings per treatment. To ensure rigorous biological replication, each treatment was divided into three independent blocks (30 seedlings per block), and awnings were independently erected for each block. After 60 days, the seedlings growth status and related parameters were comprehensively evaluated. Leaf samples were harvested and immediately immersed in liquid nitrogen for preservation. Samples were stored at -80 °C for subsequent biochemical, transcriptomic, and qRT-PCR analyses.

### Measurement of seedling growth indicators

After 60 days of shading treatments, the basal diameter, plant height, crown width, and leaf count of the seedlings were measured using precision calipers and a steel ruler. The increments in basal diameter, plant height, crown width, and leaf count were then calculated. The root characteristics of the seedlings were also analyzed after the 60-day shading period. To assess root characteristics without selection bias, three seedlings were randomly selected and harvested from each of the three biological replicates per treatment. The root length, root diameter, average root diameter, and total root surface area were measured using a root scanner (Microtek Scan Maker I800, WinRHIZO Pro).

### Determination of the chlorophyll content

Leaves from A. beshanzuensis subjected to different shading treatments were excised and homogenized. 0.2 g leaf sample was weighed and the chlorophylls were extracted 2h using 10 ml of 80% acetone until they turned completely white. The mixture was then filtered. The filtrate was transferred to a 25 mL volumetric flask and diluted to the mark with 80% acetone. All extraction and measurement steps were performed in the absence of light to prevent chlorophyll photodegradation. A blank control was prepared using 80% acetone. Absorbance was measured at 663 nm and 645 nm using a UV-Visible spectrophotometer (Purkinje General, Beijing, China). Each wavelength was measured in triplicate for accuracy. The contents of chlorophyll a (Chl a) and chlorophyll b (Chl b) were calculated using the Lambert-Beer law, as follows (Ma et al., 2023):


Chl a = (12.71A663 - 2.59A645)× V/(1000×FW)

Chl b = (22.88A645 - 4.76A663)× V/(1000×FW)

Here, A663 and A645 represent the absorbance values at 663 nm and 645 nm, respectively, V is the extraction solution volume, and FW is the leaf’s fresh weight (g).

### Determination of chlorophyll fluorescence parameters

Chlorophyll fluorescence parameters of seedlings subjected to various shading treatments were measured using a Mini-PAM fluorometer (Walz, Effeltrich, Germany) and WinControl 3 software. The procedure described by Dai et al. (2009) was followed to measure leaf chlorophyll fluorescence parameters. Before measurement, the leaves were adapted to darkness for 30 min (Yu et al., 2023; Lu et al., 2024). Under dark adaptation conditions, the needles were exposed to low-intensity measuring light to record the initial fluorescence value (Fo). Subsequently, a saturating pulse of light (8000 µmol photons m^-^² s^-^¹ for 0.8–1 second) was applied to fully close the PSII reaction centers, allowing the maximum fluorescence value (Fm) to be recorded. Based on the recorded Fo and Fm values, the gain settings of the instrument were adjusted to ensure the signal remained within the detector’s linear range, thereby preventing signal saturation or insufficient sensitivity. Subsequently, an internal light source was used to measure Yield (photochemical yield), ETR (electron transport rate), qP (photochemical quenching coefficient), and NPQ (non-photochemical quenching) under varying light intensities (Lu et al., 2026). Measurements were repeated three times for validation.

### Determination of enzyme activity

The malondialdehyde (MDA) content in the leaves was measured using the thiobarbituric acid method described by Yan et al. (2020). 0.1 g leaf sample was homogenized in 1 mL of extraction solution on ice, centrifuged at 8,000×g at 4 °C for 10 minutes, and the supernatant was collected. Absorbance at 532 nm and 600 nm was measured with a blank control, and ΔA532 and ΔA600 were calculated as the differences between sample and blank absorbance values. MDA content was calculated using the formula:


MDAcontent(nmol/g)=32.258×(ΔA532-ΔA600)/W

W: weight (g).

The activities of superoxide dismutase (SOD), catalase (CAT), and peroxidase (POD) were determined using commercial kits from Beijing Solabao Technology Co., Ltd. (Beijing, China), following the manufacturer’s instructions.

For SOD activity, leaf samples (0.1 g) were homogenized in 1 mL of extraction solution on ice, centrifuged at 8,000×g at 4 °C for 10 minutes, and the supernatant was collected. Absorbance values for A_determination_, A_control_, A1_blank_ and A2_blank_ at 560 nm were recorded. ΔA determination and ΔA blank were calculated as A_determination_ − A_control_ and A1_blank_ − A2_blank_, respectively. SOD activity was calculated using the following formulas:


$$\text{Inhibitionpercentage=}\left({\text{ΔA}}_{\text{blank}}{\text{-ΔA}}_{\text{determination}}\right){\text{/ΔA}}_{\text{blank}}\times 100\%$$

SODactivity(U/gmass)=11.4×Inhibitionpercentage/(1-Inhibitionpercentage)×W×F

W: weight (g); F: dilution ratio.

For CAT activity, the preparation of samples followed the same protocol as for SOD. Absorbance values at 405 nm for A_assay_, A_control_, A_blank_ and A_standard_ were recorded. ΔA_assay_, and ΔA_standard_ were calculated as A_assay_ − A_control_ and A_standard_−A_blank_, respectively. CAT activity was calculated as:


$$\text{CAT activity }\left(\text{U/g mass}\right){\;=\;10\times \text{(ΔA}}_{\text{standard}}{\text{-ΔA}}_{\text{assay}}{\text{/ΔA}}_{\text{standard}}\text{)/W}\times \text{F}$$

F: Sample dilution ratio; W: Sample weight (g).

For POD activity, the preparation of samples followed the same protocol as for SOD. Absorbance values at 470 nm were recorded as A_1_​ (at 30 seconds) and A_2_ (at 1 minute), and ΔA was determined as A_2_−A_1_​. POD activity was calculated using the formula:


POD activity (U/g mass) = ΔA×Vt/(W×Vm/Vs)/0.01/T = 7133×ΔA/W

Vt: Total reaction volume (1.07 mL); Vm: Sample volume added (0.015 mL); Vs: Extraction solution volume (1 mL); T: Reaction time (1 min); W: Sample weight (g).

### Determination of soluble sugar, soluble protein, and proline content

Proline content (µg/mL) was determined following the method described by Bates et al. (1973). Initially, 0.1 g leaf sample was homogenized in 1 mL of extraction solution using a homogenizer. The homogenized mixture was centrifuged at 8,000 × g for 10 min, and the supernatant was collected. The supernatant’s absorbance was measured at 520 nm using a spectrophotometer.

Soluble sugar content (µg/mL) was determined using the phenolic sulfuric acid method described by Dubois et al. (1951). 0.5 g leaf sample was weighed, mixed with water in a 1:25 ratio, and ground into a homogenate. The homogenate was extracted using ultrasonic soaking for 1 h. 1 mL leaf extract was added to 5 mL of anthracene sulfate reagent (1 g of anthracene dissolved in 1 liter of 80% sulfuric acid solution). The mixture was boiled in a water bath for 10 minutes, then cooled to room temperature. The absorbance at 620 nm was measured using a UV-Visible spectrophotometer.

Soluble protein content was quantified using the Coomassie brilliant blue G-250 method described by Liang et al. (2018). Specifically, 0.5 g sample was homogenized with water in a 1:25 (w/v) ratio. The homogenates were centrifuged at 10,000 × g for 10 minutes at 4 °C, and the supernatants were collected for further analysis. 0.1 mL leaf extract was added to 4.9 mL of Coomassie brilliant blue G-250 solution. The absorbance of the mixture was measured at 595 nm.

### RNA sequencing and RT–qPCR analysis

Total RNA was extracted using the Trizol Invitrogen Kit (CA, USA), and RNA quality and purity were assessed using the Agilent 2100 Bioanalyzer with the RNA 1000 Nano LabChip Kit (Agilent, CA, USA). Paired-end sequencing was performed on the Illumina Novaseq™6000 platform (Illumina, USA) by Lianchuan Biotech Company, which also conducted the RNA extraction and sequencing procedures.

Sequencing data underwent *de novo* assembly. CutAdapt and Perl scripts were used to remove adapter contamination, low-quality bases, and indeterminate sequences. The sequence quality was validated using FASTQC. Trinity 2.4.0 was employed for *de novo* transcriptome assembly. Transcripts were grouped into clusters based on shared sequence content, referred to as “genes.” The longest transcript in each cluster was designated as the representative “gene” sequence or unigene.

Unigene expression levels were quantified using Salmon software, and TPM values were calculated. The R package DESeq2 was used to identify statistically significant results. Differentially expressed genes (DEGs) were identified by DESeq2 based on an adjusted *P*-value<0.05 and |log_2_ fold change| ≥ 1. Metabolic pathway analysis was performed using GO (Gene Ontology, http://www.geneontology.org) and KEGG (Kyoto Encyclopedia of Genes and Genomes, http://www.genome.jp/kegg/). Raw transcriptome data were deposited in the NCBI Gene Expression Omnibus (GEO) under accession number PRJNA1063909.

For qRT-PCR analysis, cDNA was synthesized using the HiScript^®^ III All-in-one RT SuperMix Kit (Vazyme, Nanjing, China) and subjected to RT-qPCR on the QuantStudio3 Real Time PCR System (Thermo Fisher Scientific). Six differentially expressed genes shared among the four groups were randomly selected. Primers were designed using Primer Premier 6.0 software, and their sequences are listed in **Supplementary Table 1**. Primer synthesis was outsourced to Shanghai Shenggong Bioengineering Company. Due to the lack of a fully annotated genome for *A. beshanzuensis*, the actin gene from the closely related *Pinus massoniana* Lamb. served as the internal reference gene, as it has shown high stability in related conifer studies under abiotic stress (Zhao et al., 2026). Relative expression levels of the six differentially expressed genes were quantified using the 2^-ΔΔCT^ method.

### Statistical analysis

Variance analysis was performed using SPSS v21.0 software. Data analysis and visualization were conducted using GraphPad Prism 9.0. Data normality and homogeneity of variances were verified using the Shapiro-Wilk and Levene’s tests, respectively. Subsequently, a one-way ANOVA combined with Duncan’s new multiple range test was used to determine significant differences (*P*< 0.05) (Zhao et al., 2025).

## Results

### Changes in leaf growth parameters and root system under different shading conditions

**Figure 1:**
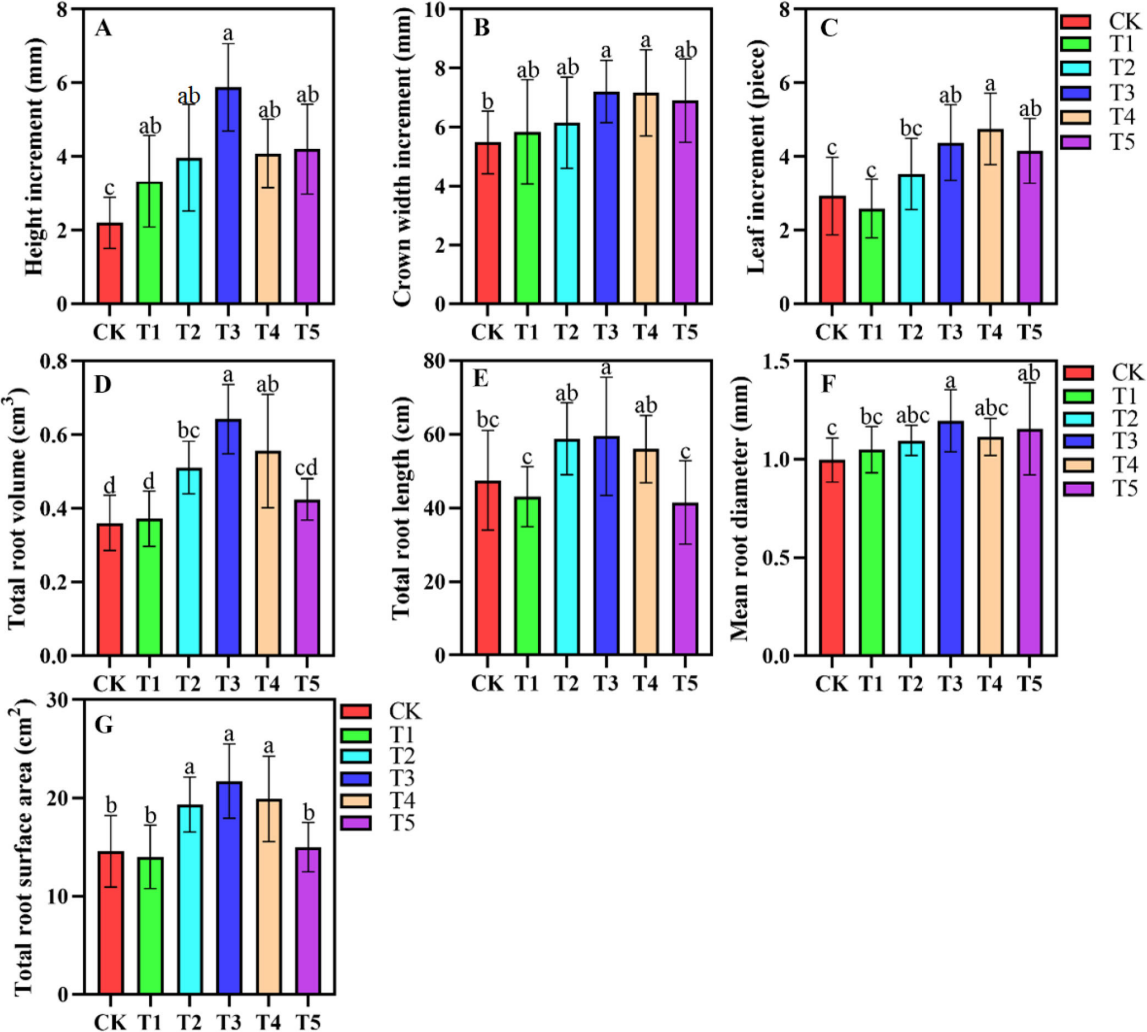
The effects of different shade treatments on the growth parameters and root system of *A. beshanzuensis*. **(A)** Height increment; **(B)** Crown width; **(C)** Leaf increment; **(D)** Total root volume; **(E)** Total root length; **(F)** Mean root diameter; **(G)** Total root surface area. Data are mean ± SD (n = 3). Different letters at the top of the bar indicate significant differences between different shading treatments (*P<* 0.05).

Shade treatments significantly influenced plant growth, including height, crown width, and leaf number (**Figure 1**). Plant height significantly increased in T1, T2, T3, T4, and T5 compared to CK. Seedling height gradually increased from T1 to T3 compared to CK. T3 resulted in the greatest seedling height, while T4 and T5 showed significantly lower growth than T3. No significant differences in plant height were observed among T2, T4, and T5 (**Figure 1A**). Crown width in CK was significantly lower than treatments (**Figure 1B**). Crown width initially increased and then decreased with increasing shade intensity, peaking in T3. Leaf number also initially increased and then decreased from T1 to T5. Leaf numbers in T2, T3, T4, and T5 were significantly higher than in CK (**Figure 1C**). As shading intensity increased, root length, total root volume, mean root diameter, and total root surface area initially increased and then decreased. T3 seedlings had the greatest total root volume, which significantly differed from CK, T1, T2, and T5. T3 seedlings also had the longest total root length, significantly differing from CK, T1, and T5 but not from T2 and T4. T3 seedlings had the largest average root diameter, significantly differing from CK and T1 but not from T2, T4, and T5. The total root surface area of T3 seedlings was the largest, significantly differing from CK, T1, and T5 but not from T2 and T4 (**Figures 1D–G**).

### Changes in leaf chlorophyll fluorescence parameters under different shading conditions

**Figure 2:**
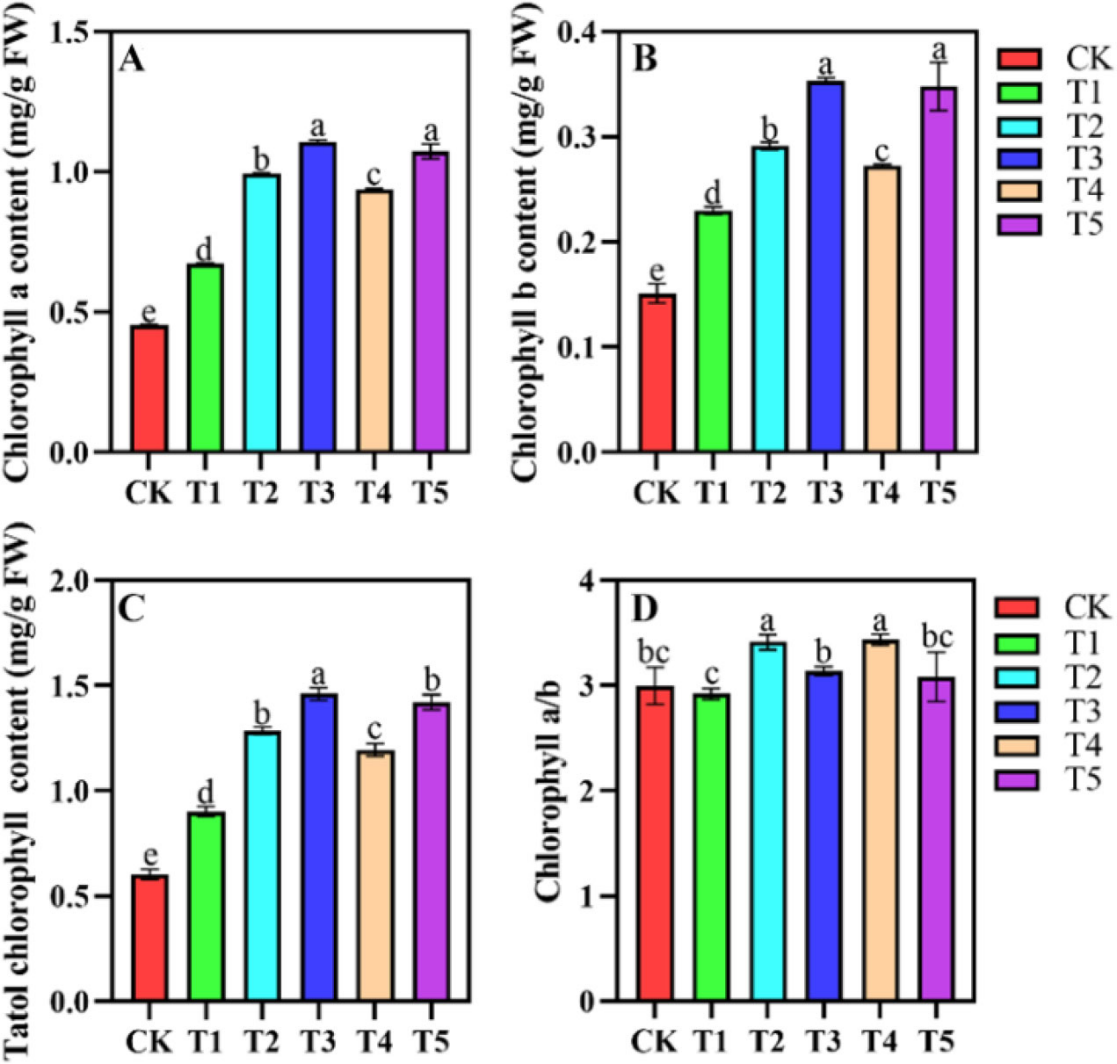
Chlorophyll a **(A)**, chlorophyll b **(B)**, total chlorophyll **(C)**, and chlorophyll a/b **(D)** concentration in *A. beshanzuensis* seedling leaves under different shading treatments. Data are mean ± SD (n = 3). Different letters at the top of the bar indicate significant differences between different shading treatments (*P<* 0.05).

**Figure 3:**
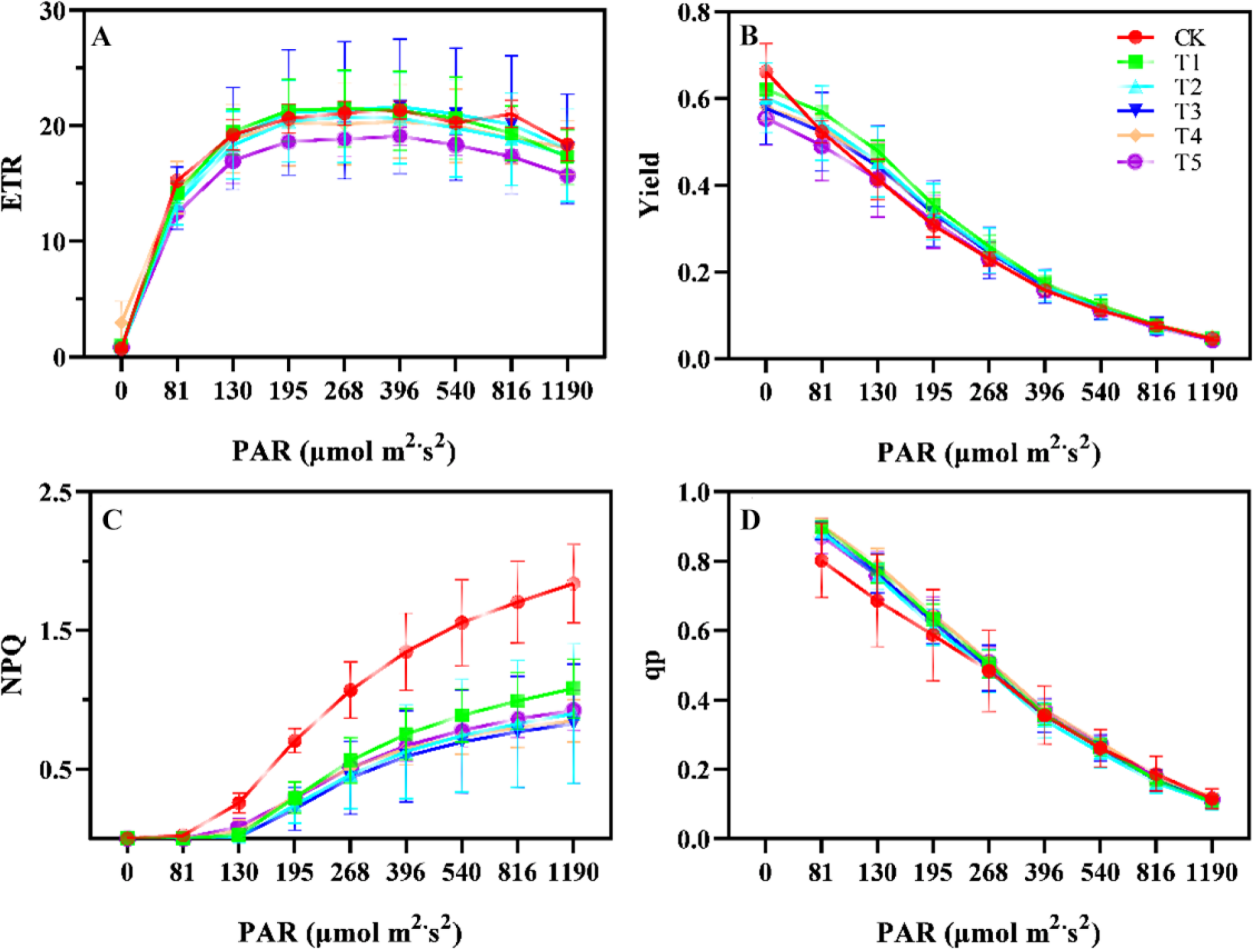
Electron transport rate (ETR) **(A)**, photosynthetic efficiency (Yield) **(B)**, non-photochemical quenching (NPQ) **(C)**, and photochemical quenching (qP) **(D)** of *A. beshanzuensis* seedling leaves under different shading treatments. Data are mean ± SD (n = 3). Different letters at the top of the bar indicate significant differences between different shading treatments (*P<* 0.05).

Shading treatments significantly influenced chlorophyll a, b, and total chlorophyll content (**Figure 2**). The highest levels of chlorophyll a, b, and total chlorophyll were observed in T3 (**Figures 2A–D**). In T3, chlorophyll a, b, and total chlorophyll concentrations increased by 141%, 140% and 232%, respectively, compared to CK. T2 and T4 had the highest chlorophyll a/b values, significantly differing from other treatments. Significant differences were observed in the physiological responses of Yield, ETR, NPQ, and qP under shading treatments (**Figure 3**). The ETR were relatively high in the CK, T1, and T3 groups, with T1 exhibiting the highest ETR within the light intensity range of 268 - 540 μmol m^-^²·s^-^¹. In contrast, T5 consistently showed the lowest ETR under all light intensities (**Figure 3A**). Under low light intensity conditions, the Yield was highest in CK, while the T5 exhibited the lowest Yield. As light intensity increased, the Yield in CK decreased significantly when the light intensity exceeded 81 μmol m^-^²·s^-^¹. The Yield of T5 remained the lowest across all light intensities (**Figure 3B**). The CK exhibited the highest NPQ, followed by the T1, whereas the NPQ values in the T2 to T5 groups were relatively low, with the T3 group showing the lowest NPQ values (**Figure 3C**). The qP of leaves in CK was lowest at light intensities below 540 μmol m^-^² s^-^¹. The qP values in T1 to T5 were relatively high, with no significant differences among these treatments (**Figure 3D**).

### Changes in leaf physiological parameters and enzyme activity under different shading conditions

**Figure 4:**
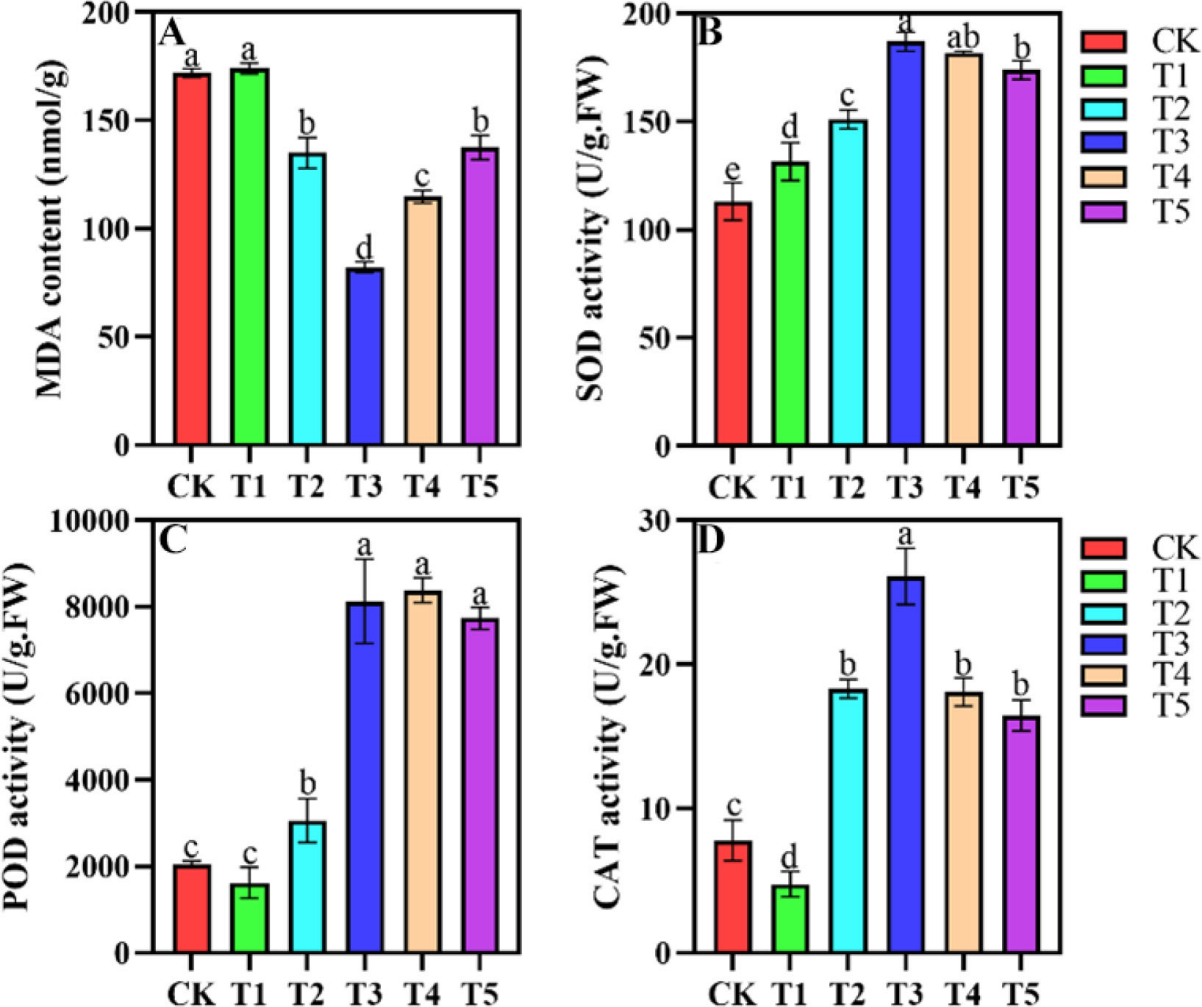
MDA content **(A)**, SOD activity **(B)**, POD activity **(C)**, and CAT activity **(D)** of *A. beshanzuensis* seedling leaves under different shading treatments. Data are mean ± SD (n = 3). Different letters at the top of the bar indicate significant differences between different shading treatments (*P<* 0.05).

Shading treatments significantly affected enzymatic activities of SOD, POD, CAT, and MDA concentration in *A. beshanzuensis* (**Figure 4**). MDA concentration in *A. beshanzuensis* leaves initially decreased and then increased with increasing shade intensity. The lowest MDA content occurred in T3, with no significant differences between CK and T1. MDA content in CK and T1 was significantly higher than in T2, T3, T4, and T5, with no significant differences between T2 and T5 (**Figure 4A**). SOD activity in *A. beshanzuensis* leaves initially increased with shading, peaking in T3, before decreasing. Compared to T3, SOD activity decreased in T4 and T5 but remained significantly higher than in CK, T1, and T2 (**Figure 4B**). POD activity showed no significant differences between CK and T1, with the lowest activity in T1. POD activity increased from T1 to T4, with higher activity in T3, T4, and T5. However, no significant differences were noted among T3, T4, and T5 (**Figure 4C**). CAT activity increased gradually from T1 to T3, with significantly higher activity in T2, T3, T4, and T5 compared to CK. CAT activity showed no significant differences among T2, T4, and T5, but was significantly higher in T3 compared to other shade treatments (**Figure 4D**).

**Figure 5:**
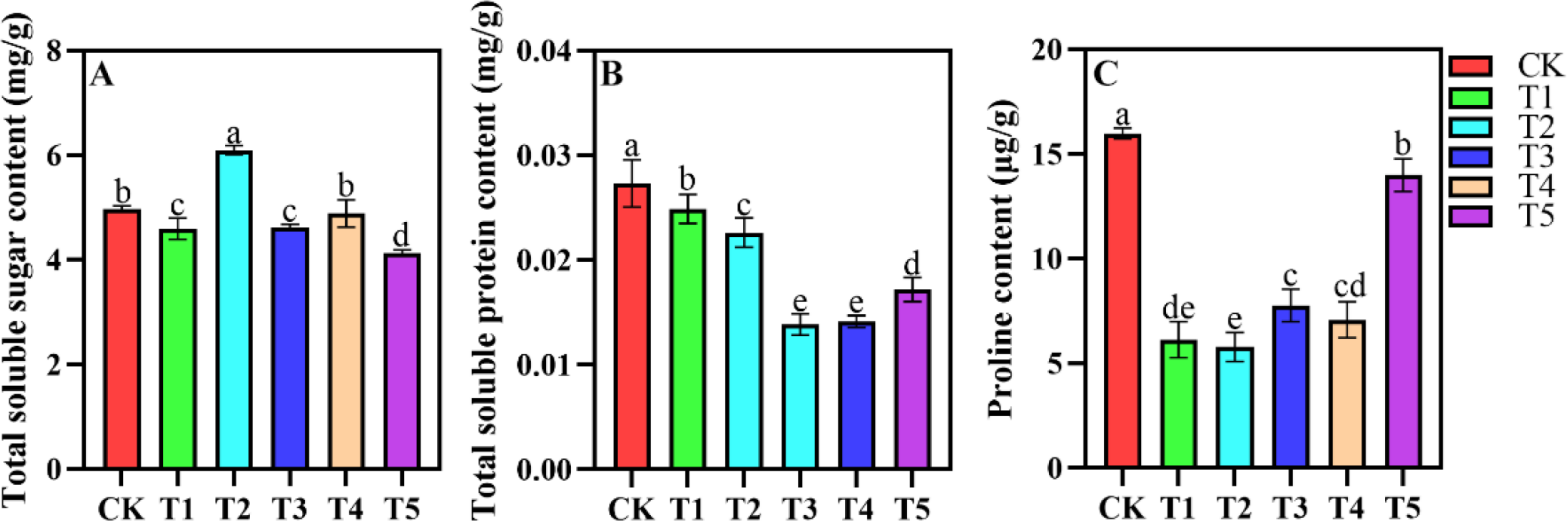
Contents of total soluble sugars **(A)**, total soluble proteins **(B)**, and proline **(C)** in *A. beshanzuensis* seedling leaves under different shading treatments. Data are mean ± SD (n = 3). Different letters at the top of the bar indicate significant differences between different shading treatments (*P<* 0.05).

Shading treatments influenced the concentrations of soluble sugars, soluble proteins, and proline content (**Figure 5**). Soluble sugar content was highest in T2, followed by CK, T1, T3, and T4, in descending order. No significant difference was found between CK and T4. Soluble sugar content in T1 and T3 was lower, with the lowest levels observed in T5 (**Figure 5A**). In *A. beshanzuensis* seedlings, soluble protein content initially decreased and then increased as shading intensity rose. No significant difference was observed between T3 and T4, with minimal soluble protein content in both groups. Soluble protein content was highest in CK, significantly differing from T1, T2, T3, T4, and T5. T1 and T2 exhibited higher soluble protein content, significantly differing from T3, T4, and T5 (**Figure 5B**). Proline content in T5 was significantly lower than in CK. Both CK and T5 exhibited significantly higher proline content compared to T1, T2, T3, and T4. Among all treatments, T2 exhibited the lowest proline content (**Figure 5C**).

### Analysis of DEGs in response to shade treatment

To elucidate the molecular responses of *A. beshanzuensis* to light-induced stress, we conducted a comprehensive transcriptome analysis using its leaf samples. We obtained 498.36 million raw reads across 12 samples, which yielded 486.34 million clean reads after filtering. The clean reads accounted for 68.07 G base pairs from four experimental groups (CK, T1, T3, and T5) with biological replicates. Sequencing reads exhibited high quality, with Phred scores showing Q20 between 97.91% and 98.12% and Q30 between 93.5% and 94.05%. The transcriptome data showed an average GC content of 45.48% (**Supplementary Table 2**). Rigorous quality control confirmed the high quality of the transcriptome dataset. Pearson correlation and PCA analyses showed significant differences among the four sequenced groups (CK, T1, T3, and T5) and consistent intra-group patterns across biological replicates. PCA identified the primary sources of variation, with PC1 and PC2 accounting for 51.79% and 33.8% of the total variance, respectively (**Supplementary Figure 1**). Significant transcriptomic alterations were observed in shading treatments (T1, T3, and T5) compared to CK (**Supplementary Figure 2**). Specifically, 324 upregulated and 422 downregulated DEGs were identified in T1 vs CK, 589 upregulated and 1282 downregulated DEGs in T3 vs CK, and 1174 upregulated and 1722 downregulated DEGs in T5 vs CK. To validate the accuracy of the RNA-Seq data, we randomly selected six genes for qRT-PCR analysis. The results demonstrated that the expression profiles of these DEGs were consistent with the RNA-Seq data, confirming the reliability of the RNA-Seq results (**Supplementary Figure 3**).

**Figure 6:**
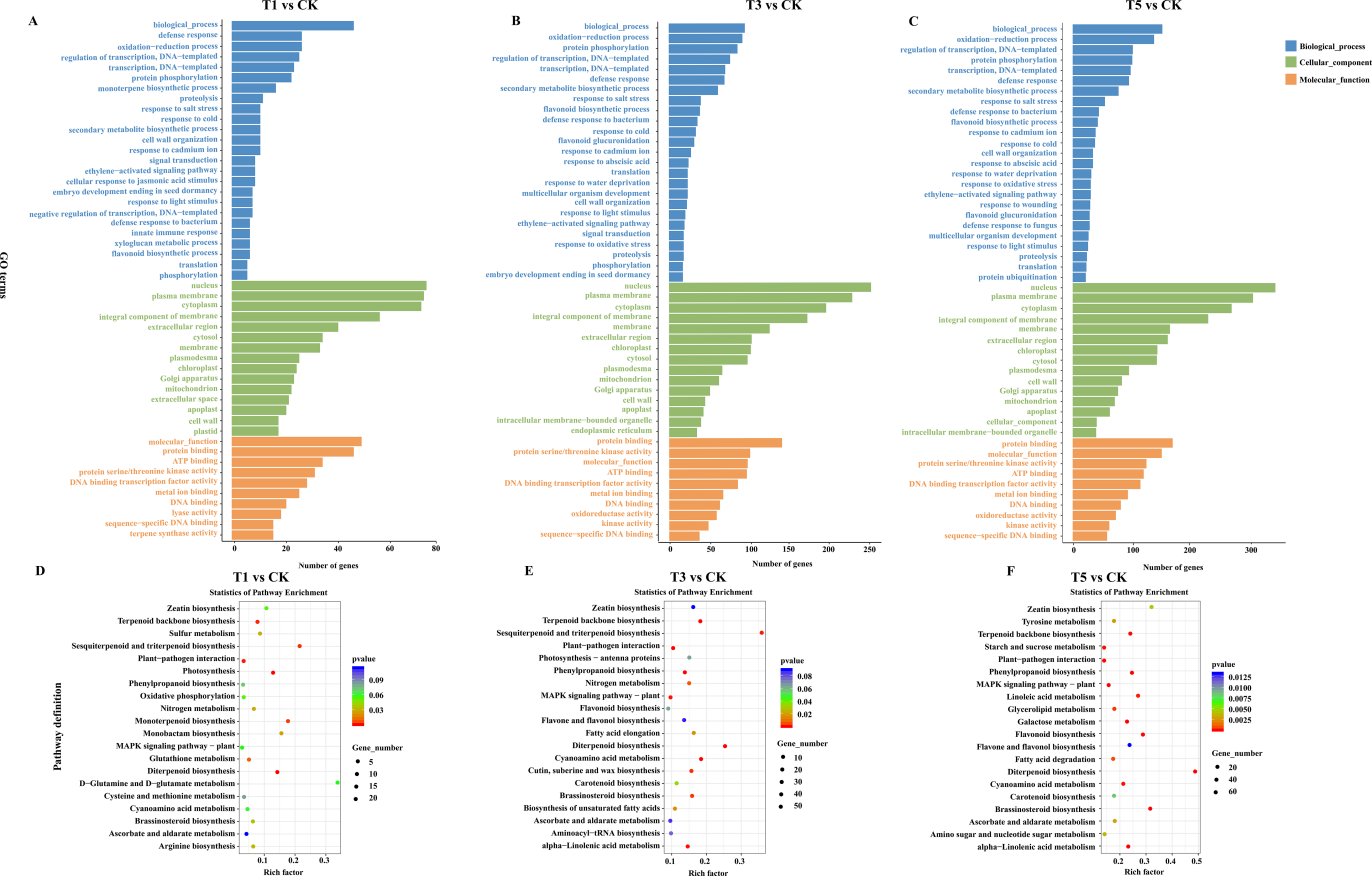
GO **(A–C)** and KEGG **(D–F)** analyses of differentially expressed genes in leaves of *A. beshanzuensis* under different degrees of shade treatments.

To elucidate the biological functions of DEGs under shading treatments, we performed GO enrichment analysis on DEGs compared to CK (**Figures 6A–C**). DEGs from T1, T3, and T5 treatments were enriched in defense responses, oxidation-reduction processes, transcription regulation, and protein phosphorylation. For cellular components, DEGs in all treatments were associated with the cell nucleus, plasma membrane, cytoplasm, integral membrane components, and chloroplast (**Figures 6A–C**). Regarding molecular functions, DEGs from T1 were enriched in protein binding, ATP binding, and serine/threonine kinase activity compared to CK (**Figure 6A**). DEGs from T3 were enriched in protein binding, serine/threonine kinase activity, ATP binding, and DNA-binding transcription factor activity (**Figure 6B**). Similarly, DEGs from T5 were enriched in protein binding, serine/threonine kinase activity, ATP binding, and DNA-binding transcription factor activity (**Figure 6C**).

Similar to GO enrichment, KEGG enrichment pathways of DEGs under various conditions were identified. Compared to CK, the top 20 enriched metabolic pathways of DEGs in T1 included terpenoid skeleton biosynthesis, plant-pathogen interaction, photosynthesis, MAPK signaling, glutathione metabolism, and sesquiterpenoid biosynthesis (**Figure 6D**). In T3, DEGs were enriched in plant-pathogen interaction, phenylpropanoid biosynthesis, MAPK signaling, sesquiterpenoid biosynthesis, carotenoid biosynthesis, photosynthesis, and carbon fixation (**Figure 6E**). In T5, enriched pathways of DEGs included starch and sucrose metabolism, plant-pathogen interaction, phenylpropanoid biosynthesis, MAPK signaling, glycerophospholipid metabolism, flavonoid biosynthesis, photosynthesis, and carbon fixation (**Figure 6F**).

### Shading affects photosynthesis in *A. beshanzuensis*

**Figure 7:**
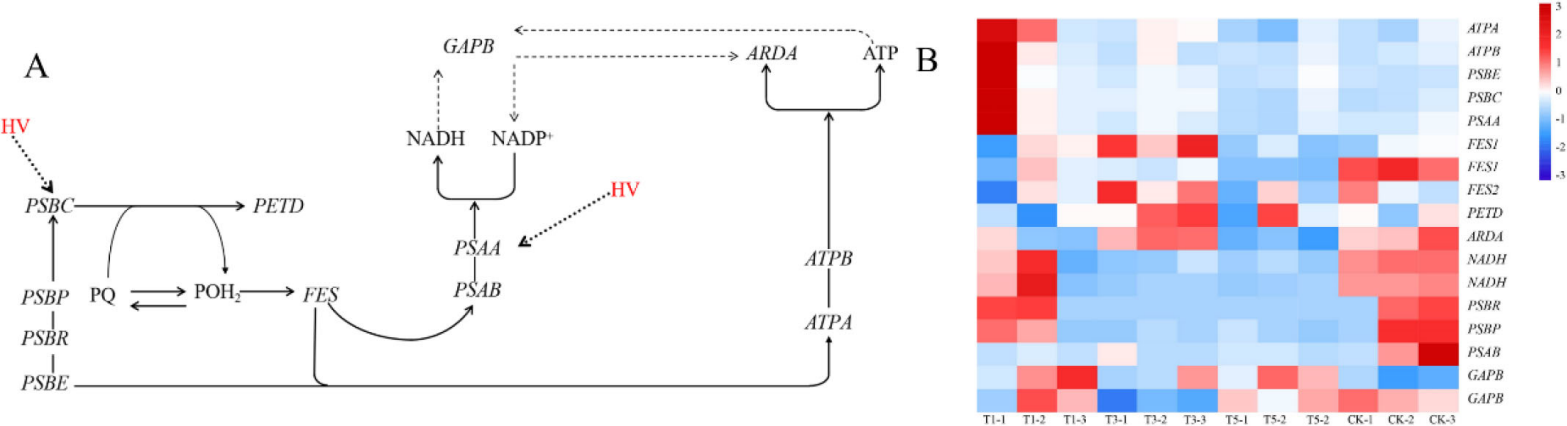
Expression patterns of photosynthesis-related genes in *A. beshanzuensis* under shading treatment. **(A)** Schematic representation of the Photosynthesis pathway. **(B)** Heatmap analysis of log_2_-fold change.

Photosynthesis played a pivotal role in plant biomass accumulation. Shading treatments influenced DEGs related to photosynthesis in *A. beshanzuensis* (**Figure 7**). In T3 and T5, shading downregulated two NADH and two PSII genes (*PsbR* and *PsbP*), while altering the expression of one *PsbB* and one *GAPB* genes. Additionally, T1 showed upregulation of two ATP synthase genes (*ATPA* and *ATPB*) and three PSII genes (*PsbA*, *PsbE*, and *PsbC*) (**Figure 7B**; **Supplementary Table 3**). In T3, *FES1*, *FES2* and *PETD* genes were upregulated. The results demonstrated that moderate shading increased plant energy accumulation.

### Shading affects defense/detoxification related genes in *A. beshanzuensis*

**Figure 8:**
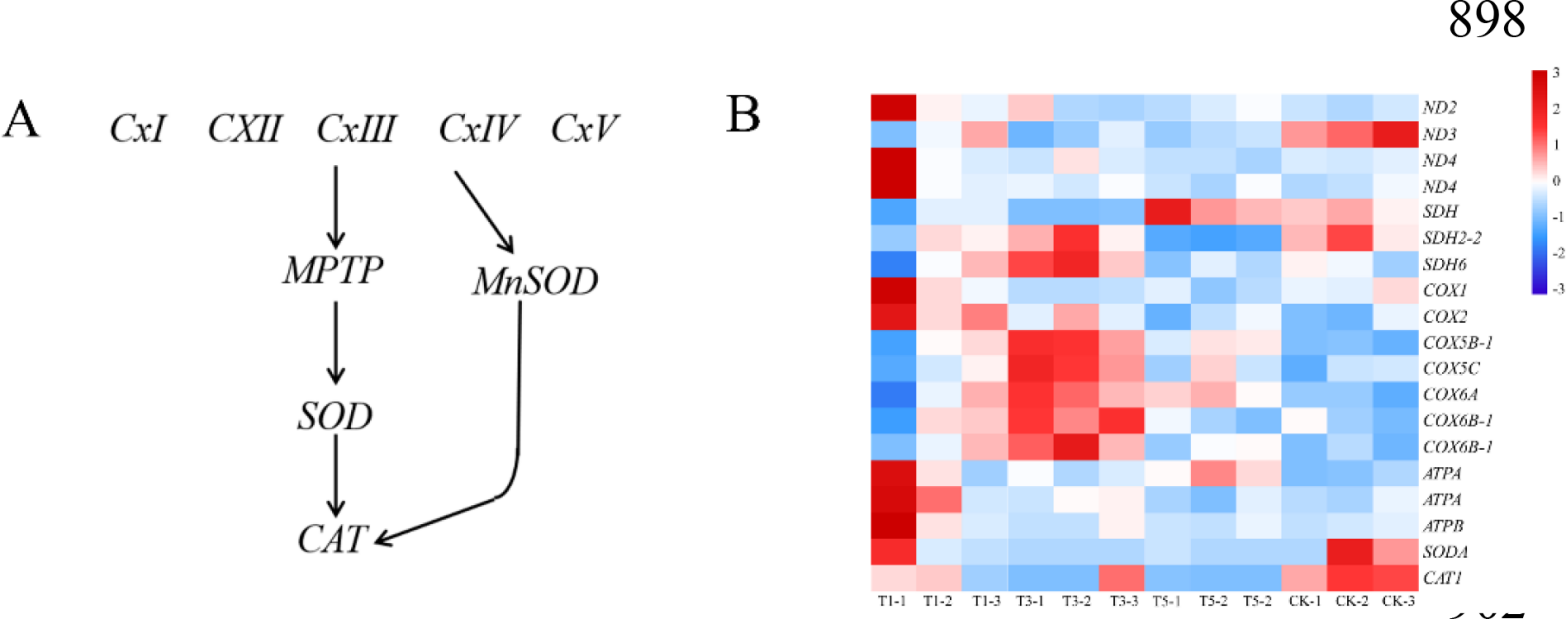
Expression patterns of ROS-related genes in *A. beshanzuensis* under shading treatment. **(A)** Schematic representation of the ROS pathway. **(B)** Heatmap analysis of log_2_-fold change.

We analyzed the expression patterns of DEGs associated with antioxidant enzymes. Shading treatments (T1, T3, and T5) enhanced the expression of *NADH dehydrogenase* (*ND2*), *Cytochrome C oxidase*, and ATP synthesis. Additionally, one *SOD* and one *CAT* genes were downregulated in T1, T3, and T5 (**Figure 8**; **Supplementary Table 4**). The findings indicated that plants activated antioxidant enzymes to adapt to light-induced stress.

### Shading affects hormone biosynthesis in *A. beshanzuensis*

**Figure 9:**
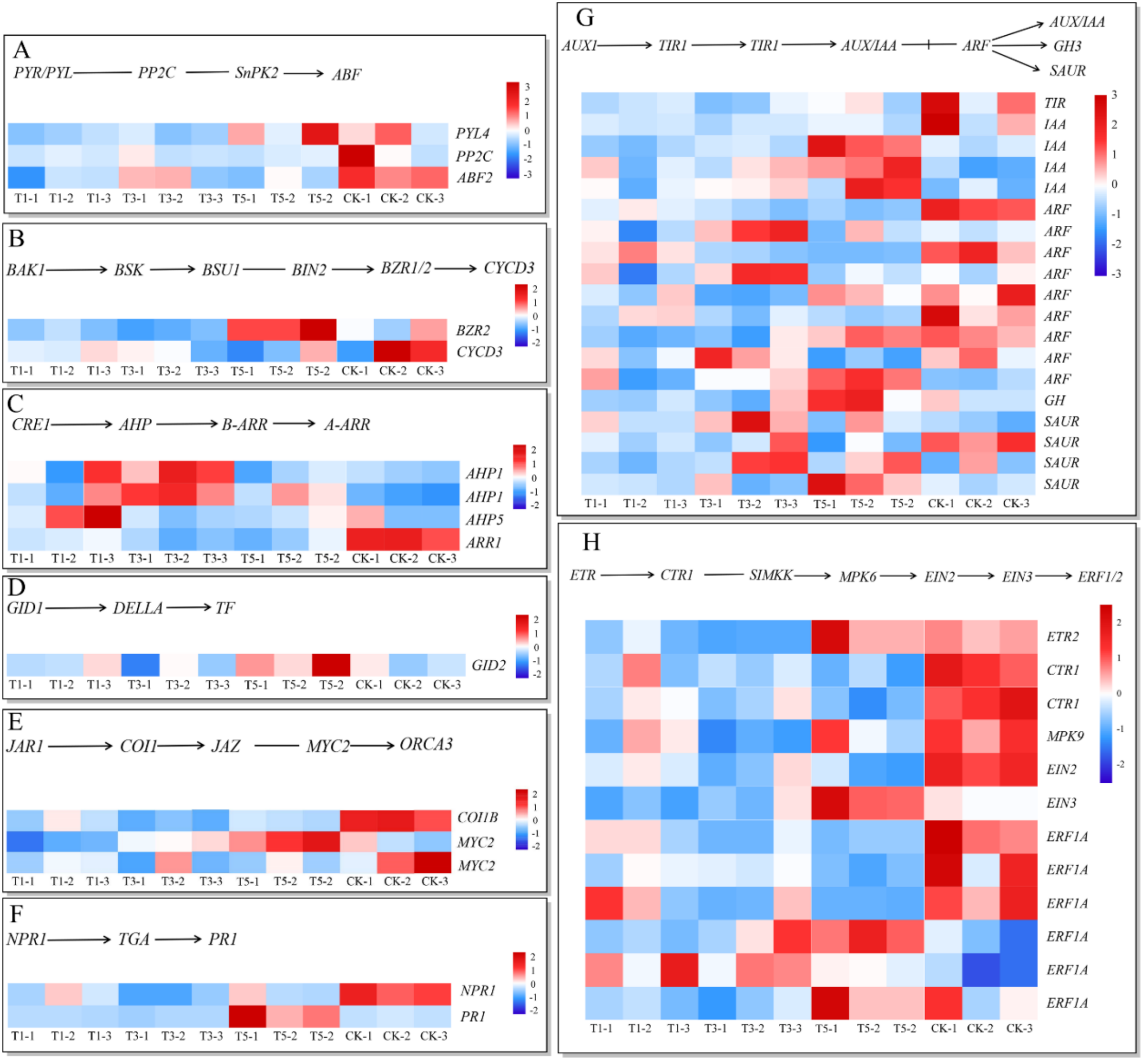
Pathway schematic diagram of hormone signal transduction-related genes in *A. beshanzuensis* under shade treatment and heatmap analysis of log_2_-fold changes. **(A)** Carotenoid biosynthesis; **(B)** Brassinosteroid biosynthesis; **(C)** Zeatin biosynthesis; **(D)** Diterpenoid biosynthesis; **(E)** Linolenic acid metabolism; **(F)** Phenylalanine metabolism; **(G)** Tryptophan metabolism; **(H)** Cysteine and methionine metabolism.

To examine the impact of shading treatments on hormone signaling pathways in *A. beshanzuensis*, we analyzed related gene expression profiles (**Figure 9**; **Supplementary Table 5**). In the ABA pathway, shading treatments downregulated one *PYR/PYL*, one *PP2C*, and one *ABF* genes (**Figure 9A**). In the BR pathway, shading reduced the expression of *CyCD3* and increased the expression of one *BZR1/2* gene in T5 (**Figure 9B**). In the cytokinin pathway, shading downregulated one type-A *ARR* and upregulated three *AHP* genes (**Figure 9C**). In the GA pathway, light stress downregulated the *GID2* in T1 and T3, while its expression was upregulated in T5 (**Figure 9D**). In the JA pathway, shading treatments downregulated one *Coi1* and one *MYC2* genes, while upregulating *MYC2* expression in T5 (**Figure 9E**). In the SA pathway, shading downregulated one pathogenesis-related gene (*NPR1*), while upregulating one pathogenesis-related protein gene (*PR-1*) in T5 (**Figure 9F**). In the auxin pathway, shading treatments downregulated several genes, including *TIR1*, most *AUX/IAA*, a majority of *ARF*, one *GH3* and specific *SAUR* genes (**Figure 9G**). In the ethylene signaling pathway, shading suppressed several genes, including one *ETR*, two *CTR1*, one *MKP*, one *EIN2* and three *ERF1/2* genes. In contrast, T5 showed upregulation of one *EIN3* and two *EBF1/2* genes (**Figure 9H**). These findings indicated that shading treatments modulated plant growth and development by altering hormone biosynthesis.

## Discussion

### Effect of shading on inhibits *A. beshanzuensis* seedlings growth

Photosynthesis served as a crucial biochemical process by which plant generates energy (Kitajima and Butler, 1975; Wang et al., 2019). The photosynthetic parameters of *A. beshanzuensis* were investigated following shading treatments. Previous studies suggested that substantial ROS accumulation under high light conditions inhibited chlorophyll synthesis, reduced the number of chloroplasts in leaves, and promoted their degradation, ultimately reducing the photosynthetic rate (Yang et al., 2022). This phenomenon was evident in our study. Additionally, moderate shading (T3) significantly enhances the electron transport rate and improves photosynthetic efficiency. In contrast, excessive shading (T5) markedly reduces the photochemical efficiency and electron transport capacity of leaves. These results aligned with findings from *Cunninghamia lanceolata* (Dong et al., 2015). These results indicate that shading treatments enhanced the proportion of open reaction centers in PSII, enabling more light energy to drive photosynthetic electron transfer and improve photosynthetic efficiency (Gao et al., 2020).

The GO terms revealed that the T3 was enriched in cellular components such as the nucleus, plasma membrane, cytoplasm, and chloroplast. These components are involved in various biological processes, including photosynthesis. Cell polarity expansion and development begin with cell wall loosening, which is facilitated by increased cell wall extensibility. Additionally, cell proliferation represents another critical mechanism driving changes in leaf morphology (Madan et al., 2021). Compared with the T1 and T5, T3 showed enrichment of more cyclins and serine/threonine kinases. Cyclins bind and regulate cyclin-dependent kinases (CDKs), which are primary regulators of cell proliferation (Novikova et al., 2013). This suggests that under optimal shading conditions, *A. beshanzuensis* seedlings experience continuous cell proliferation, resulting in favorable leaf growth.

The KEGG pathway analysis showed that DEGs in shade treatment involved photosynthesis-related pathways, including photosynthesis, photosynthesis-antenna proteins, carbon assimilation, and carotenoid metabolism. For the “photosynthesis” and “photosynthesis-antenna protein” pathways, DEGs were significantly upregulated in both the T1 and T3 compared with the CK. This repression of photosynthesis-related genes under excessive shading indicates a negative impact on the photosynthetic activity of *A. beshanzuensis* seedlings. Carotenoids, as accessory pigments, play a crucial role in photoprotection under high light intensities (Saeed et al., 2015). They assist chloroplasts in capturing wavelengths of light that chlorophyll cannot absorb while also dissipating excess energy from excited chlorophyll molecules (Dall'Osto et al., 2012). This prevents lipid peroxidation and reduces damage to thylakoid membranes, which are highly susceptible to oxidative stress (Muller et al., 2001). In this study, compared with the control group, DEGs associated with the “carotenoid metabolism” pathway were enriched and significantly downregulated in T3 and T5, indicating a reduction in photooxidative stress in seedlings subjected to shading.

DEGs related to the PSI and PSII systems were identified. The PSI system encompassed various ferredoxins. Among these, *PsaA*, a key PSI component, alleviated photoinhibition during PSI repair or recombination, as reported by Wada et al. (2021). Consequently, upregulation of the *PsaA* gene stabilized PSI and delayed reductions in photosynthetic efficiency (Zhou et al.2021). Drought-stressed plants exhibited increased PsaA protein levels, enhancing resilience to adverse environments, as observed by Dahal and Vanlerberghe (2018). Additionally, salt stress impaired energy transfer in PSI by inhibiting *PsaA* expression (Suo et al., 2020; Tokarz et al., 2021). Our results confirm this. *PsaB* served as a critical photosensitive component of PSI. Previous research showed that saline-alkali stress suppressed *PsaB* expression, reducing photosynthetic function (Qian et al., 2009). T3 and T5 significantly downregulated the expression of *PsaB*. PSII is the primary reaction center in photosynthesis, responsible for capturing light energy and converting it into chemical energy, which is subsequently used to generate ATP. PSII is the primary reaction center in photosynthesis, responsible for capturing light energy and converting it into chemical energy, which is subsequently used to generate ATP. PsbR and PsbP are two extrinsic subunits of the oxygen-evolving complex associated with PSII in higher plants, where PsbP binding to PsbR is essential for optimal oxygen-evolving activity (Liu B et al., 2022). While the absence of PsbP is typically associated with decreased PSII quantum yield under standard conditions (Guo et al., 2022). Our findings revealed a significant downregulation of *PsbP* and *PsbR* under T3 and T5. This downregulation likely reflects a structural reorganization of the PSII antenna complex to adapt to lower photon flux and prevent over-excitation under shaded conditions. Despite the downregulation of these specific PSII components, our physiological data showed T3 maintained high ETR and improved overall photosynthetic efficiency. This suggests a compensatory mechanism whereby the concurrent upregulation of critical downstream electron transport and energy accumulation genes, such as *FES1*, *FES2*, and *PETD*, observed in the T3 treatment, likely stabilizes the coupling between PSI and PSII. Consequently, *A. beshanzuensis* is able to maintain robust electron transfer and adaptively balance energy assimilation under moderate light restriction.

### Effect of shading on defense/detoxification related enzymes and genes

Light played a crucial role in photosynthesis in plants, while excessive shading adversely impacted plant growth and development. Our findings indicated that an optimal shading intensity of approximately 45% was ideal. Deviations from this optimal light intensity, both above and below, adversely affected plant biomass accumulation, growth, and development. Environmental stress led to ROS accumulation in plant cells, resulting in cell damage and lipid peroxidation in the membrane (Cheng et al., 2021). MDA, a stable end product of lipid peroxidation, served as a crucial indicator of free radical-induced damage to plant cell membranes under various environmental stress conditions (Xu et al., 2024), as corroborated by our study. Under CK, T1 and T2 treatments, *A. beshanzuensis* were subjected to strong light stress, membrane lipid peroxidation was enhanced, MDA content was increased, and protective enzyme activity was low. In T4 and T5, under low light stress, MDA was higher than T3, and protective enzyme activity was lower than T3. The data suggests that intense light stress exceeds the plant defense mechanisms, resulting in significant impairment of the synthesis rates or activities of antioxidant enzymes, thereby hampering plant growth (Lu et al., 2017). However, our study did not find a complete correspondence between antioxidant enzyme activity levels and their gene expression levels. The researchers noted that no strictly linear relationship existed between them (Kim et al., 2021). Thus, further investigation into the relationship between enzyme activity and gene regulation was warranted. Furthermore, abiotic stress increased the concentrations of soluble sugars, soluble proteins, and proline, functioning as a protective mechanism for plants (Liu et al., 2018). Our observations revealed that shading treatment triggered a marked increase in soluble sugar content in the leaves of *A. beshanzuensis*. Moderate shading can enhance the accumulation of soluble sugars, thereby improving the plant’s adaptability to environmental stress (Li et al., 2024a), whereas strong light and excessive shading weakened this tolerance.

SOD and CAT, along with other enzyme protection systems, constituted crucial mechanisms for plant cells to counteract oxidative damage. They effectively mitigated cellular damage induced by various physicochemical stressors, prevented ROS accumulation, and preserved the integrity of cell membrane structure. The activity of antioxidant enzymes relied on the stress tolerance of plants and varied among species (Szymanska et al., 2017). In this study, exposure to intense light decreased the activities of SOD, POD, and CAT, downregulated gene expression, and resulted in unfavorable leaf growth conditions. Analogous observations were recorded in the investigation conducted by Lu et al. on tomatoes (Lu et al., 2017). Moderate shading enhanced the production rate and functionality of antioxidant enzymes, which benefited plant growth and development. The present study revealed that as shading intensity escalated, both SOD and CAT initially increased before declining, suggesting that optimal shading treatments might augment plant resistance (Huang et al., 2022). Additionally, shading increased cytochrome c oxidase activity (Lu et al., 2022). In our study, the expression of genes associated with cytochrome c oxidase was up-regulated under shading, thereby reducing the damage caused by excessive ROS to *A. beshanzuensis*.

### Effect of different light intensities on phytohormone pathways

Prior studies postulated that shading modulated physiological and biochemical mechanisms by upregulating ABA-related genes (PYL4, PP2CA, and APF3), significantly enhancing plant tolerance (Asghar et al., 2020, 2022; Kim et al., 2022). However, our findings diverged from this hypothesis. The discrepancy could be attributed to the unsuitability of sunlight conditions for the growth and development of A. beshanzuensis, necessitating shading treatment to optimize its habitat. Furthermore, auxin modulated plant growth under abiotic stress by activating the antioxidant system (Guilfoyle and Hagen, 2007). Auxin is perceived through a co-receptor complex consisting of TIR1/AFB receptors and AUX/IAA proteins. This perception leads to the ubiquitination and degradation of AUX/IAA proteins, thereby releasing their inhibition on ARFs. This process plays a crucial role in the plant response to changes in light conditions (Peer, 2013; Lv et al., 2021). Under shading treatment, the downregulation of one TIR and five ARF genes suggests that light may directly or indirectly modulate plant growth by altering auxin biosynthesis and/or signaling pathways. Furthermore, SUAR genes were significantly upregulated in the T3 and T5 treatments. SAUR expression was negatively correlated with auxin biosynthesis (Kant et al., 2009). Therefore, the reduction in auxin content in A. beshanzuensis may be associated with the upregulation of SUAR genes under shading treatment. Collectively, these observations suggested that shading stress impeded signaling pathways in A. beshanzuensis, including those related to ABA and auxin. In the BR pathway, the shading induced downregulation of CyCD3 and upregulation of BZR2 under T5 conditions highlight a finely tuned regulation of cell division and elongation processes. CyCD3, a crucial promoter of cell cycle progression, was suppressed, suggesting reduced cellular proliferation under shading (Zhu et al., 2024). This suppression may reflect an adaptation to prioritize resource allocation for survival over active proliferation. The upregulation of BZR2, which enhances cell elongation, could act as a compensatory mechanism to maintain growth and structural integrity (Li et al., 2023), enabling A. beshanzuensis to adapt to reduced light availability effectively.

In the JA signaling pathway, the active molecule jasmonate-isoleucine (JA-ILE) bound to Coi1, released MYC2, and regulated various plant hormones as well as growth and development (Li et al., 2024b). In our results, Coi1 and MYC2 were downregulated under most shade treatments, while MYC2 was upregulated during the T5 phase. This upregulation possibly represented a critical threshold response, enabling plants to achieve a balanced distribution of growth and defense processes under moderate light restriction. Shading led to a downregulation of ARR and an upregulation of AHP genes in the cytokinin pathway. ARR acts as a negative regulator of cytokinin signaling (Ai et al., 2024), while AHPs are positive regulators. This opposing regulation suggests a rebalancing of cytokinin signaling, potentially enhancing plant growth in response to shading. The differential regulation of GID2 across treatments (downregulated in T1 and T3, upregulated in T5) highlights the dynamic response of the GA pathway to varying shading intensities. GID2 is a key regulator of GA signaling, promoting the degradation of DELLA proteins that suppress growth (Razzaq and Du, 2024). The upregulation of GID2 in T5 suggests enhanced GA signaling, possibly promoting stem elongation to reach light sources.

Ethylene receptors regulated the growth and development of plants, with ETR, EIN, and CTR acting as negative regulators in the ethylene signaling pathway (Dubois et al., 2018; Shi et al., 2016). A decrease in the expression of ETR and CTR1 hindered the growth and development of plants (Tao et al., 2015). Members of the Ethylene Response Factor (ERF) gene family played a pivotal role in influencing plant responses to abiotic stressors (Candar-Cakir et al., 2016). In the ethylene signaling pathway, shading suppressed several key genes, including ETR, CTR1, MKP, EIN2 and ERF1A, indicating a general downregulation of ethylene signaling under shaded conditions. Ethylene is known to regulate stress responses and senescence, and its suppression may delay aging processes and prioritize growth. Notably, under T5 conditions, the upregulation of EIN3 and ERF1A suggests a compensatory mechanism to fine-tune ethylene-mediated responses. This dynamic adjustment might help balance growth and stress adaptation under moderate shading.

## Conclusion

In this study, we conducted an in-depth analysis of the regulatory mechanisms governing the response of A. beshanzuensis to shading treatments. Our findings revealed that shading treatments, particularly medium shading treatment (T3), significantly enhanced growth metrics, including plant height, crown width, total root volume, total root length, average root diameter, total root surface area, and chlorophyll-a, chlorophyll-b, and total chlorophyll concentrations. Medium shading treatment has been shown to enhance the ETR, thereby improving the photosynthetic efficiency of plants. Moderate shading treatment boosted biomass production in A. beshanzuensis, fostering its optimal growth and development. These shading treatments also increased the activities of SOD, POD, and CAT enzymes, which reduced excessive ROS accumulation. Transcriptomic analysis revealed that the expression levels of FES1, FES2, and PEYD genes were significantly upregulated under T3, potentially contributing to enhanced photosynthetic activity. Furthermore, moderate shading treatment dynamically modulated the expression of genes related to auxin, ABA, and ethylene signaling pathways, often suppressing stress-induced ethylene responses to prioritize structural growth. These findings indicate that utilizing 45% shading nets during artificial nursery breeding, and selecting appropriately shaded microhabitats for wild reintroductions, will be critical strategies for the successful conservation of this critically endangered species.

## Data Availability

The datasets presented in this study can be found in online repositories. The names of the repository/repositories and accession number(s) can be found below: https://www.ncbi.nlm.nih.gov/, PRJNA1063909.
