# Supplementary material for: Regulating growth and stress responses in Abies beshanzuensis seedlings: integrated impacts of shading on morphology, physiology, and molecular pathways
Source: Front Plant Sci. 2026 May 13;17:1833397. doi: 10.3389/fpls.2026.1833397 (PMC13212522; doi:10.3389/fpls.2026.1833397)
Supplement: Supplementary file 2 [file Table2.docx]

**Regulating growth and stress responses in *Abies beshanzuensis* seedlings: integrated impacts of shading on morphology, physiology, and molecular pathways**

Hongfei Lu^a,^ ^b^, Zhen Pang^b^, Chuting Yan^a^, Rongguang Lan^c^, Lifang Zhang^c^, Sumei Wu^c^, Yougui Wu^c^, Xiaoyi Li^b^, Likang Zhao^b, *^, Mingjian Yu^d, *^

**^a^** Dongyang Wood Carving Industry Innovation Research Institute, Zhejiang Guangxia Construction Vocational and Technical University, Dongyang, Zhejiang 322100, China

^b^ College of Life Science, Zhejiang Sci-Tech University, Zhejiang Province Key Laboratory of Plant Secondary Metabolism and Regulation, Hangzhou 310018, China

^c^ Qianjiangyuan-Baishan National Park Qinyuan Conservation Center, Qingyuan 323800, China

^d^ College of Life Science, Zhejiang University, Hangzhou 310018, China

*Corresponding Author.

a15237268212@163.com

fishmj@zju.edu.cn

**Supplemental files**

**Supplemental tables** (see EXCEL file)

**Table S1. Primers sequence of differentially expressed genes in leaves of *A. beshanzuensis* seedlings under different shade treatments**

**Table S2. Overview of leaf sequencing quality control of *A. beshanzuensis* seedlings under different degrees of shade treatments**

**Tbale S3. Expression of genes involved in photosynthesis**

**Tbale S4. Expression of genes involved in defense/detoxification**

**Tbale S5. Expression of genes involved in phytohormone biosynthesis**

**Supplemental figures**

**Fig. S1** (A) Differential gene expression analysis between groups. (**B**) Pearson correlation coefficient heat map of leaves of *A. beshanzuensis* seedlings under different shade treatments. (**C**) Principal component analysis of different treatment groups, with different colors representing different treatments.

**Fig. S2** Volcano plot analysis of Differentially Expressed Genes (DEGs) under different shading treatments. (A) Volcano plot of DEGs between T1 and CK; (B) Volcano plot of DEGs between T3 and CK; (C) Volcano plot of DEGs between T5 and CK.

**Fig. S3** RT-PCR verified the differentially expressed genes of leaves of *A. beshanzuensis* seedlings under different degrees of shade treatment. The bar graph is the relative expression measured by qRT-PCR; the broken line graph is the gene expression obtained by RNA-seq. A: T1; B: T3; C: T5; D: CK.

**
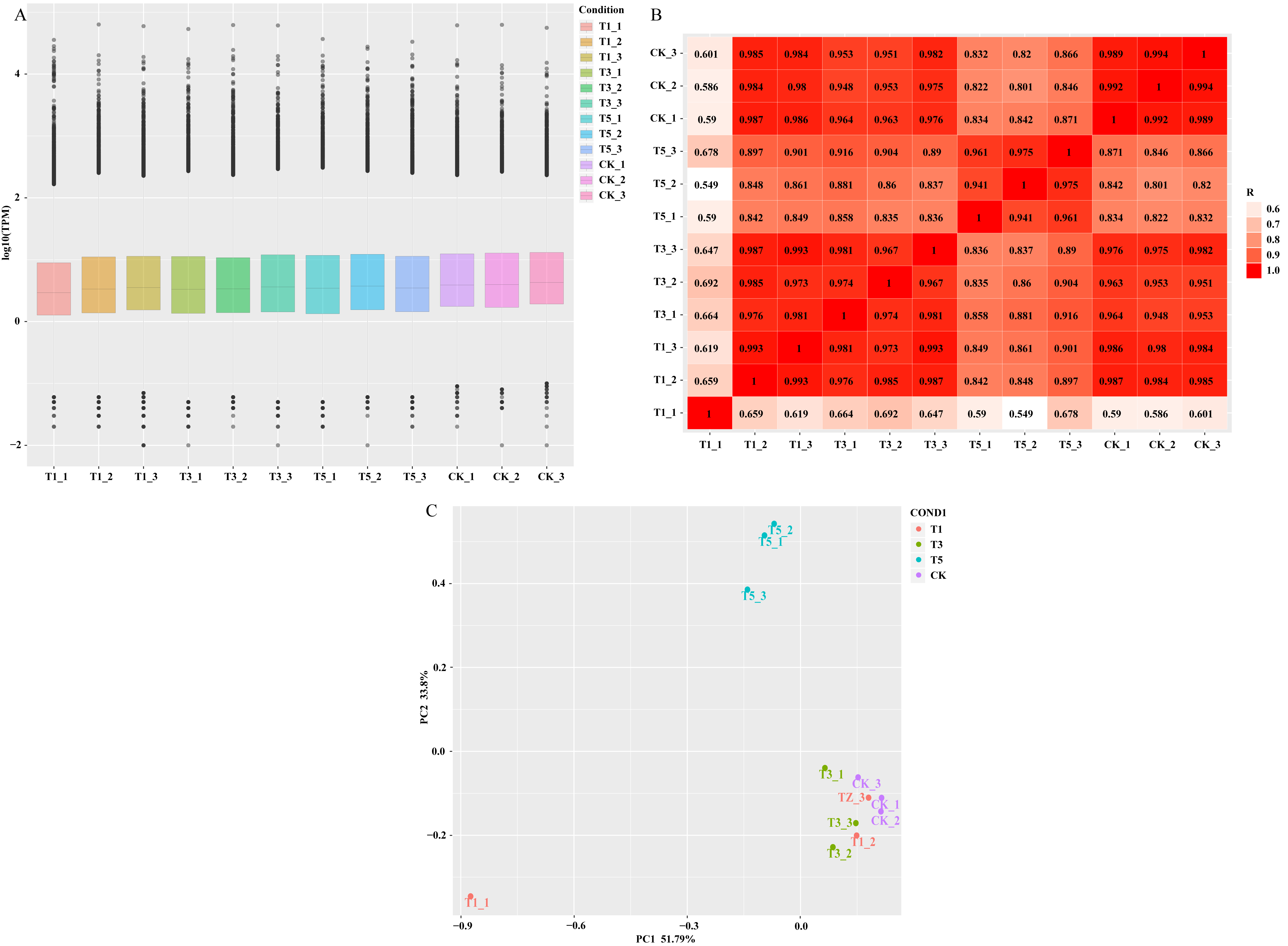
Fig. S1** (A) Differential gene expression analysis between groups. (**B**) Pearson correlation coefficient heat map of leaves of *A. beshanzuensis* seedlings under different shade treatments. (**C**) Principal component analysis of different treatment groups, with different colors representing different treatments.

**
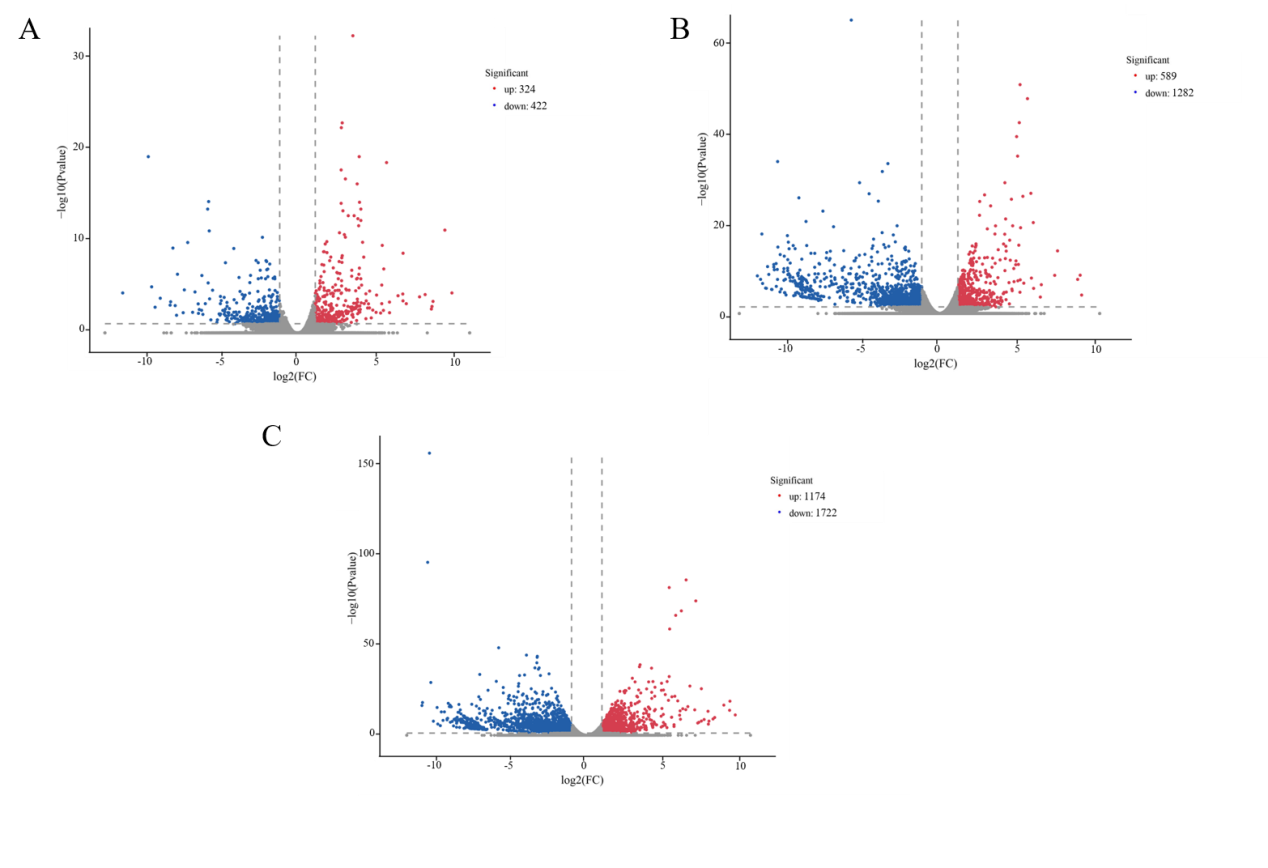
Fig. S2** Volcano plot analysis of Differentially Expressed Genes (DEGs) under different shading treatments. (A) Volcano plot of DEGs between T1 and CK; (B) Volcano plot of DEGs between T3 and CK; (C) Volcano plot of DEGs between T5 and CK.

**
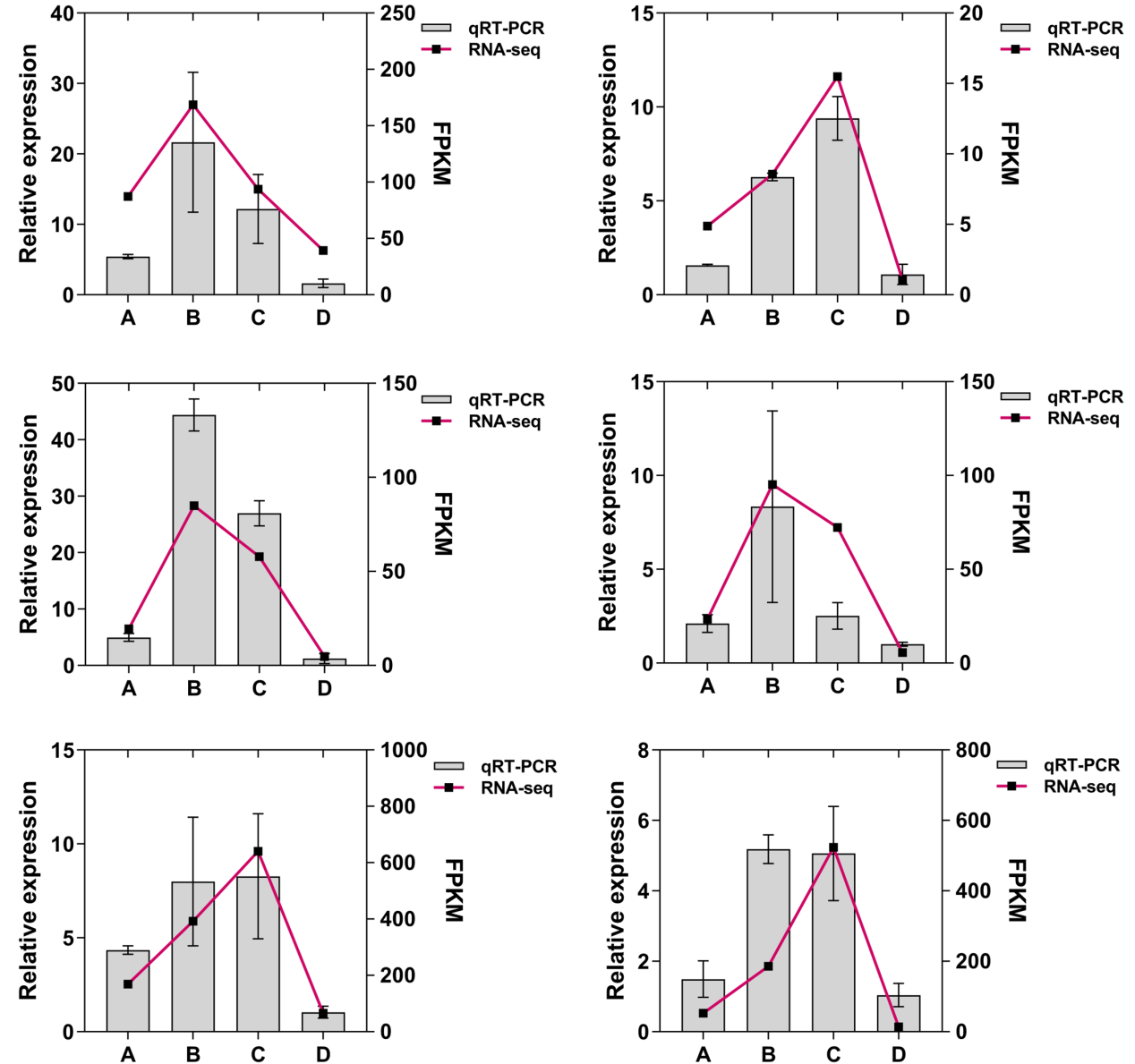
Fig. S3** RT-PCR verified the differentially expressed genes of leaves of *A. beshanzuensis* seedlings under different degrees of shade treatment. The bar graph is the relative expression measured by qRT-PCR; the broken line graph is the gene expression obtained by RNA-seq. A: T1; B: T3; C: T5; D: CK.
